# Diagnostic criteria in proliferative verrucous leukoplakia: Evaluation

**DOI:** 10.4317/medoral.19424

**Published:** 2014-03-08

**Authors:** Begoña García-Chías, Laura Casado-De La Cruz, Germán C. Esparza-Gómez, Rocío Cerero-Lapiedra

**Affiliations:** 1 Department of Medicine and Buccofacial Surgery, Faculty of Dentistry, Complutense University of Madrid

## Abstract

Objectives: to evaluate the ability of the diagnostic criteria proposed by Cerero et al in 2010 to perform an early diagnose in patients with proliferative verrucous leukoplakia. Study Design: retrospective study with patients diagnosed with leukoplakia at Oral Medicine Service at Oral Medicine and Surgery Department at Dentistry Faculty at Universidad Complutense of Madrid. Results: the criteria were applied in 116 patients, turning positive in 40 cases. Out of these, 24 (60%) had been previously diagnosed with PVL. Most frequent criteria were major criteria A and E, concerning lesion’s site and histopathology, and minor criteria b and c, concerning sex and smoking habit. Conclusions: diagnostic criteria developed by Cerero et al can be a useful tool for an early diagnose of PVL, as in 60% of the cases, the criteria would have allowed to make an early diagnose of the disease.

** Key words:**Proliferative verrucous leukoplakia, criteria, diagnose, early.

## Introduction

Oral cancer is among the ten most common cancers worldwide, although there is a wide geographical variation in the incidence (1,2). A significant percentage of oral cancers are preceded by potentially malignant disorders of the oral mucosa, including leukoplakia. Leukoplakia is defined as “a white plaque of questionable risk, having excluded (other) known diseases or disorders that carry no increased risk for cancer” (3).

In 1985 Hansen et al described proliferative verrucous leukoplakia (PVL) as a long-term progressive condition, which develops initially as a white plaque of hyperkeratosis that eventually becomes a multifocal disease (4). The lesions are slow growing and persistent, as well as irreversible and resistant to all forms of treatments, with a high recurrence rate (up to 70%). Throughout its development is common to find erythematous and/or verrucous areas that occasionally progress to verrucous carcinoma or squamous cell carcinoma (5).

Usually, PVL diagnosis is made according to Hansen’s first definition in 1985, not taking into account the latter ones. The inconvenience is that this diagnosis is usually made once the lesions have evolved, and so they have a less favourable prognosis. Therefore, it would be important to be able to diagnose early the disease, in order to identify susceptible patients and treat them more exhaustively (6).

There are two previous studies, one by Ghazali *et al.* and another by Gandolfo *et al*, that tried to develop a set of diagnostic criteria to their respective cases, although these are just a transcription of Hansen’s definition (7,8). Cerero *et al.* considered that these criteria were not enough, and proposed another set of criteria based not only in Hansen’s definition but also in various findings reported in latter published cases of PVL. These criteria can be divided in major and minor criteria: (6)

1. Major Criteria (MC)

a. A leukoplakia lesion with more than two different oral sites, usually gingiva, alveolar ridge and palate.

b. Presence of a verrucous area.

c. The lesions have spread or engrossed during the development of the disease.

d. There has been a recurrence in a previously treated area.

e. Histopathologically, we can find from simple epithelial hyperkeratosis to verrucous hyperplasia, verrucous carcinoma or squamous cell carcinoma, in situ or infiltrating.

2. Minor Criteria (mc)

a. An oral leukoplakia lesion that occupies at least 3 cm when adding all the affected areas.

b. The patient is a woman.

c. The patient (male or female) does not smoke.

d. Disease evolution longer than 5 years.

In order to establish the diagnosis of PVL, it was suggested that two of the following combinations have to exist: three major criteria (being E among them) or two major criteria (being E among them) plus two minor criteria (6).

This set of diagnostic criteria has not been used and evaluated in any case series, and therefore we do not know their real utility and effectiveness. Thus, our objective is to apply them in a set of patients with oral leukoplakia and evaluate their capacity to perform a correct early diagnose of PVL. Our second objective will be to analyze the clinical characteristics of the cases with positive criteria.

## Material and Methods

-Patients: in this study we included all patients with a clinical diagnosis of leukoplakia and a histopathological diagnosis that did not exclude leukoplakia. This was a retrospective study that analyzed all patients with this pathology who were treated at Oral Medicine Service, Faculty of Dentistry at Universidad Complutense of Madrid between 1984 and 2011.

-Method: The criteria were applied in all the patients. The clinical and histopathological data were obtained from their medical records. The first author carried out this review manually. Criteria were assessed at the time of the leukoplakia diagnosis, one year, and five years later. A score of 2 points was given for each major criterion (MC), and 1 point for each minor criterion (mc).

If the patient had six or more points in their first visit at the Oral Medicine Department, they would become part of group 1. Later, we would analyse the data obtained when applying again the criteria in the annual and five-years check-up, and we would check the evolution. If the patient did not obtain 6 points in the first visit, we would check the data at annual check-up, so that they would become part of group 2. The same process would be done if the patient did not meet the requirements in the annual check-up. We would check the data of 5 years after the first diagnosis, and those who had six or more points at that moment would became part of group 3. Finally, we would check the evolution.

Also, some clinical data from patients in group 1, 2 and 3 were recorded: age, sex, tobacco habit, follow-up period, number of lesions, locations, clinical type, presence of pain, biopsies, recurrence of the lesions.

## Results

First of all we selected from the Oral Medicine Service Database all those patients who had a clinical diagnosis of leukoplakia. We obtained 146 patients, but the inclusion criteria led to the selection of 116 patients.

Therefore, the diagnosis criteria set described by Cerero *et al.* was applied in 116 patients. Criteria were positive in 40 patients, distributed as follows: 21 had positive criteria right in the moment they were diagnosed with leukoplakia (group 1), 8 had positive criteria in the annual check-up (group 2), and 11 at the five-years check-up (group 3). In every case we observed that the criteria would stay the same or increase over time.

Of these 40 patients with positive criteria, 24 (60%) had been previously diagnosed with PVL, relying on Hansen’s definition. Out of these 24, 18 (75%) obtained positive criteria in the moment they were diagnosed with leukoplakia or in the annual check-up. It is possible that the other 16 patients that have not been diagnosed as PVL yet, are on early stages of the disease ( Table 1).

**Table 1:**
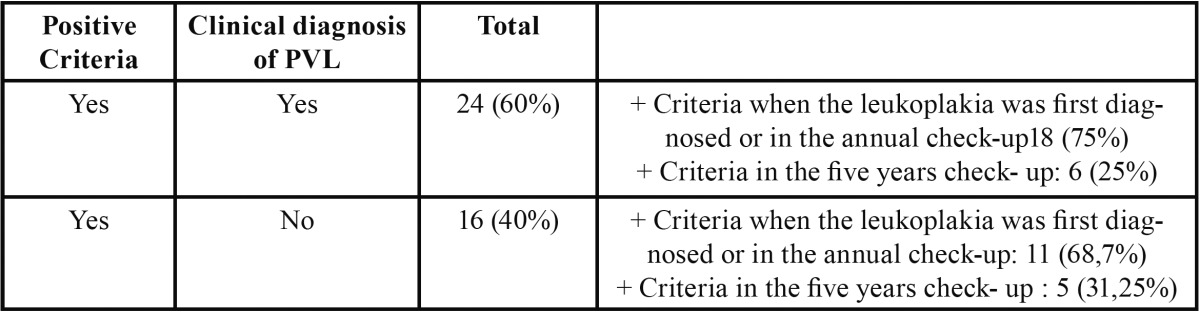
Positive criteria: patients that meet the diagnostic criteria. PVL: proliferative verrucous leukoplakia.

Sensitivity (0.88) and specificity (0.82) of these criteria was calculated, as well as the positive predictive value (0.6) and negative predictive value (0.96), ( Table 2).

**Table 2:**
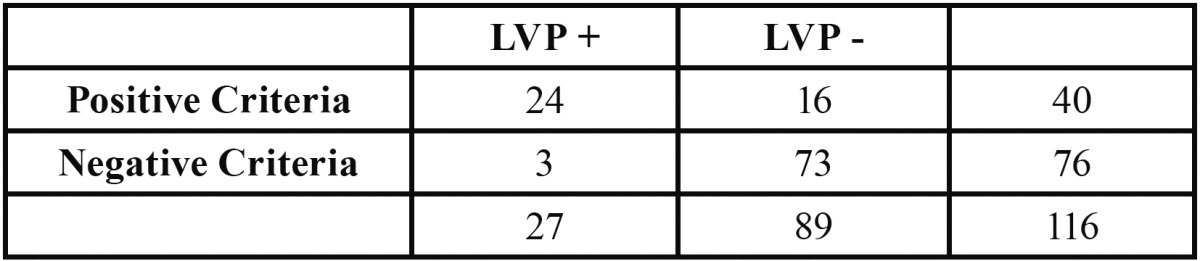
Sensibility, specifity, positive and negative predictive value data.

Most frequent criteria were major criteria A and E, concerning lesion’s location and histopathology, and minor criteria b and c, concerning sex and smoking habit, ( Table 3).

**Table 3:**
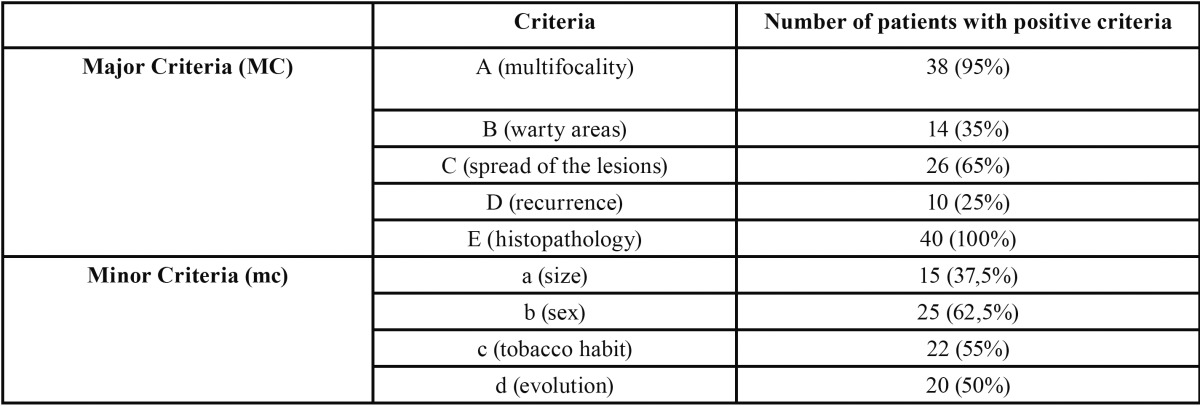
Patients with positive criteria.

Among all patients with positive criteria we can find more women (62,5%). Average age is 62,3 years, and there are more non-smoking patients (67,5%%). There is an average of 4,4 lesions per patient, being gingiva and alveolar ridge the most common locations, followed by buccal mucosa. Homogeneous lesions were found in 39 patients, while 16 had also verrucous lesions and 9 eritroleukoplakia lesions. In 22 patients lesions were bigger than 3 cm2 and only 5 patients showed pain. Number of biopsies varied from 1 to 6; in every case there was hyperkeratosis and 50% of them showed signs of dysplasia. Three patients had verrucous carcinoma and four patients had oral squamous cell carcinoma. The follow-up period average was 44 months. The most common treatment was surgical removal of the lesions, with recurrences in 50% of the cases.

## Discussion

Proliferative verrucous leukoplakia is considered an independent entity from oral leukoplakia, as there are differences in its clinical and histopathological characteristics, as well as a more aggressive evolution. The malignant transformation rate of this pathology can appear in up to 70% of the patients, and some studies report even 100% of malignant transformation (5,9). That is why an early diagnose can lead in a better prognosis for this kind of patients.

In previously published case series, authors used to make PVL diagnosis according to Hansen’s definition. Even though this first definition is quite accurate, it has not been updated in almost 30 years. In 2010 Cerero *et al.* proposed a set of diagnosis criteria, based not only in Hansen’s definition, but also in case series published later (6).

Diagnosis criteria proposed by Cerero et al. consist on 9 criteria, in order to help the clinician to establish a suspected diagnosis of PVL as early as possible so we can have a better prognosis.

First criterion refers to multifocality of the lesions, which had already been described by Hansen. In our study, 38 out of 40 patients with positive criteria had lesions in at least 2 different locations, with a media of 4,4 lesions per patient. More commonly affected areas were gingiva and alveolar ridge, followed by buccal mucosa. These results are similar to those published by other authors (4,5,10).

Major criteria B and C refer to lesions evolution from first diagnosis. Our results show that 11 patients had verrucous lesions when we first diagnosed them. In 26 patients the lesions spread or engrossed during the follow up period.

About major criterion D, we found recurrence of surgical removed lesions in at least 50% of the cases. This number is lower than those showed by other authors, with percentages up to 80%.

The last major criterion, E, is essential in order to confirm PVL diagnosis. It is a very wide criterion that tries to reflect from a histopathological point of view the natural evolution of the disease (6). In our study only seven patients have developed so far some kind of carcinoma. Three of them had lesions that evolved to verrucous carcinoma and four to oral squamous cell carcinoma. The average time to appearance of the carcinoma was almost 4 years (3,77 years).

About minor criterion, the first one refers to the leukoplakia lesions size. In PVL, Cerero *et al.* consider that the presence of multiple lesions in different sites is more relevant than the size of the lesion itself and therefore, they consider these criteria as a minor one. In our study, 55% of the patients had lesions bigger than 3 cm2 when adding all the affected areas. Second minor criterion refer to patient’s sex. Most PVL studies report a higher prevalence of this pathology in women; we can even find a 4:1 ratio (4,5,11). Other studies show fewer differences between sexes, but always in smaller case series (9,12).

There are also discrepancies among different authors regarding tobacco habit. Although there is a consensus about tobacco not having a significant influence on the disease, there is a big variety in the percentages we find in different studies. This could be related to the decision to consider or not former smokers as smokers. In our study, we included former smokers for more than 10 years as non-smoker patients, basing our decision in several previous studies that state that after that time, former smoker patients have the same odds of suffering oral cancer than non-smoker patients (13). In our study, 18 patients where smokers or former smokers for less than 10 years, and therefore did not meet criterion c. This percentage (45%) is similar to those shown by other authors, like Gandolfo *et al.* (37%) (8).

Finally, we should consider the disease evolution, as PVL is considered a long-term pathology. Even though it is complicated to determine the exact moment when the lesions appeared, most of case series published report that evolution period is longer than 5 years.

Recently Carrad *et al.* (2013) published a critical appraisal of these diagnostic criterion (14). In their opinion, major criteria A should not include keratinized mucosa as the most frequently involved site in PVL, but considerer that any site of the oral cavity may be involved. However, in our study, and in all but one of the case series included in Cerero *et al.* study, we found that most of the lesions would appear in keratinized mucosa (gingiva, alveolar ridge and palate). About major criterion E, we consider that even though that histopathology description is not limited to PVL, it can be useful to discard different pathologies, and therefore it should be maintained as diagnostic criterion. About the higher prevalence in women and the absence of tobacco habit should be kept in our opinion as minor criterion, because even if there are not definitive data about this, we can find important differences among percentages found in leukoplakia case series and PVL case series (15). Finally, we consider that the modified diagnostic criteria can be useful to diagnose a PVL that is already in an advance stage, but maybe they are not enough if we want to make an early diagnose, and foresee if a single leukoplakia has the risk to develop PVL.

Out of our 40 patients that met the diagnostic criterionproposed by Cerero *et al.* 24 of them (60%) had a previous diagnose of PVL. In all of them we found that the lesions spread and enlarged during their development. Out of these 24 patients, 18 of them met the diagnostic criteria either on the first time they came to our clinic or in the annual check-up. Therefore, we consider that these criteria may be useful to make an early diagnose of PVL.

In the remaining patients the lesions have not changed in the successive check-ups, but we cannot assure that they will not develop PVL in the future. In our opinion, the fact that 40% of patients that met the diagnostic criteria did not develop PVL, does not detract the criteria utility, since its implementation is simple and non-traumatic, and allow us to make a more appropriate track to our patients.

We found interesting the presence of both lichen planus and PVL lesions associated in this kind of patients. This make us think about the possibility of not making a correct differential diagnosis between these two pathologies or maybe about the coexistence of the lesions (16).

In conclusion, we consider that the diagnostic criteria developed by Cerero *et al.* allow us to make an early diagnose of PVL, so that 60% of the patients that meet the criteria end up developing the disease. Most common criteria when the first diagnose is made are major criterion A and E and minor criterion b and c. Thereby, we consider that the clinician should suspect of having a patient with PVL when having a patient that is a non-smoker with lesions in multiple locations and with an histopathological diagnosis compatible with leukoplakia. Likewise, it is necessary to assess the presence of warty lesions, lesions that have thickened and spread over time, and lesions with a long-term evolution.
